# *ALKBH5* regulates chicken adipogenesis by mediating *LCAT* mRNA stability depending on m^6^A modification

**DOI:** 10.1186/s12864-024-10537-2

**Published:** 2024-06-25

**Authors:** Xiaohuan Chao, Lijin Guo, Chutian Ye, Aijun Liu, Xiaomeng Wang, Mao Ye, Zhexia Fan, Kang Luan, Jiahao Chen, Chunlei Zhang, Manqing Liu, Bo Zhou, Xiquan Zhang, Zhenhui Li, Qingbin Luo

**Affiliations:** 1 State Key Laboratory of Livestock and Poultry Breeding, South China Agricultural University, Guangzhou, China; 2 College of Animal Science, South China Agricultural University, Guangzhou, China; 3 College of Animal Science and Technology, Nanjing Agricultural University, Nanjing, China

**Keywords:** MeRIP-seq, Chicken, Adipogenesis, *ALKBH5*, *LCAT*, Stability

## Abstract

**Background:**

Previous studies have demonstrated the role of N6-methyladenosine (m^6^A) RNA methylation in various biological processes, our research is the first to elucidate its specific impact on *LCAT* mRNA stability and adipogenesis in poultry.

**Results:**

The 6 100-day-old female chickens were categorized into high (*n* = 3) and low-fat chickens (*n* = 3) based on their abdominal fat ratios, and their abdominal fat tissues were processed for MeRIP-seq and RNA-seq. An integrated analysis of MeRIP-seq and RNA-seq omics data revealed 16 differentially expressed genes associated with to differential m^6^A modifications. Among them, *ELOVL* fatty acid elongase 2 (*ELOVL2*), pyruvate dehydrogenase kinase 4 (*PDK4*), fatty acid binding protein 9 (*PMP2*), fatty acid binding protein 1 (*FABP1*), lysosomal associated membrane protein 3 (*LAMP3*), lecithin-cholesterol acyltransferase (*LCAT*) and solute carrier family 2 member 1 (*SLC2A1*) have ever been reported to be associated with adipogenesis. Interestingly, *LCAT* was down-regulated and expressed along with decreased levels of mRNA methylation methylation in the low-fat group. Mechanistically, the highly expressed *ALKBH5* gene regulates *LCAT* RNA demethylation and affects *LCAT* mRNA stability. In addition, *LCAT* inhibits preadipocyte proliferation and promotes preadipocyte differentiation, and plays a key role in adipogenesis.

**Conclusions:**

In conclusion, *ALKBH5* mediates RNA stability of *LCAT* through demethylation and affects chicken adipogenesis. This study provides a theoretical basis for further understanding of RNA methylation regulation in chicken adipogenesis.

**Supplementary Information:**

The online version contains supplementary material available at 10.1186/s12864-024-10537-2.

## Background

Adipose tissue is widely regarded as the major site of lipid deposition and lipid metabolism. Adipose tissue has energy storage, endocrine and metabolic functions, it affects the health, metabolic balance and immune homeostasis in humans and animals [1–5]. A number of metabolic diseases caused by obesity have become an increasing global health problem [6, 7]. Similarly, excessive deposition of abdominal fat in chickens can also lead to metabolic diseases and increase feed wastage in poultry, adding enormously to the cost of poultry production.

Adipogenesis is a complex process with transcription factors at the core, coordinated with epigenetic factors and transcriptional cofactors together to form a complex and programmed transcriptional regulatory network [8–10]. Epigenetic modification plays an important role in fat formation and deposition [11–14]. In addition to DNA methylation and histone modification, RNA methylation has been implicated in the regulation of adipogenesis [15–18]. In 3T3L1 cells, *Zfp217* is involved in the regulation of overall m^6^A modification by activating the transcription of the m^6^A demethylase *FTO*. This suggests that *Zfp217* plays an important role in the generation and differentiation of fat [19]. Similarly, studies conducted b demonstrated that *FTO* enhances RNA stability in chicken preadipocytes in an m^6^A-dependent manner, thereby increasing *CTNNB1* expression. Furthermore, m^6^A has been demonstrated to promote the translation of HIF1A mRNA and to regulate the browning and thermogenesis of white fat cells [20]. However the mechanism of *LCAT* mRNA methylation on abdominal adipogenesis in poultry is unclear.

The MeRIP-seq technique employs m^6^A-specific antibodies to enrich M6A-modified RNA fragments, thereby facilitating the analysis of m^6^A modifications and RNA epigenetic modifications within the transcriptome range [21]. We aim to identify the mRNA of interest by MeRIP-seq to further explore the regulatory mechanism of m^6^A methylation in chicken adipogenesis. This study is the first to clarify the role of *LCAT* m^6^A methylation during adipogenesis. Lecithin cholesterol acyltransferase (*LCAT*) is a key protein in the peripheral high-density lipoprotein metabolism pathway, which has been reported to be mainly involved in cholesterol reverse transport and energy metabolism [22–24]. To date, *LCAT* plays an important role in atherosclerosis and is associated with abdominal subcutaneous fat and abdominal visceral fat [25–28]. We observed that both *LCAT* expression and its mRNA methylation were downregulated in adipose tissue in the low-fat group. *ALKBH5*, an m^6^A demethylase, was found to regulate mRNA fate through m^6^A demethylation and has been implicated in the biological regulation of several cancers [29, 30]. *ALKBH5* has been reported to affect transcription and translation by regulating mRNA fate through m^6^A demethylation [31, 32]. *ALKBH5* has been demonstrated to be involved in the pathogenesis of osteoarthritis by influencing the stability of *CYP1B1* mRNA through m^6^A demethylation [33]. Furthermore, studies have demonstrated that *ALKBH5* regulates the energy metabolism of haematopoietic stem cells and progenitors through RNA stability, which is mediated by m^6^A modification [34]. In addition, studies have shown that *ALKBH5* is associated with obesity and can regulate the proliferation and differentiation of chicken adipocyte progenitors [35, 36]. Using qPCR, we found that *ALKBH5* was differentially expressed between the high-fat group and the low-fat groups. To our knowledge, no study has shown how *ALKBH5* mediates LCAT demethylation to influence adipogenesis in chickens. The purpose of this study was to investigate the potential m^6^A modification sites using sequencing data and impact of *LCAT* mRNA demethylation on chicken adipogenesis by cell biology. This can help to enhance our understanding of the regulatory mechanism of RNA methylation in chicken adipogenesis.

## Methods

### Animal testing and ethical statements

The animals provided by Guangdong Wens for this study were six female Sanhuang chickens, and the single cage feeding was carried out after the growing stage, and pellets were fed twice a day. At 100 days of age, they were euthanised by dislocation of the first cervical vertebra, and their abdominal fat tissues were collected, weighed and stored in liquid nitrogen. The body weights and abdominal fat weights of the 6 experimental animals are listed in Table 1. The animal experiments were approved by the Animal Care Committee of South China Agricultural University (SCAU#2,017,015; September 13, 2017).

**Table 1:** Abdominal fat percentage of 6 subjects

Sample	weight (g)	Abdominal fat weight (g)	Abdominal fat rate (%)
L1	1576.2	61.8	3.92
L2	1550	56.8	3.66
L3	1360	50.3	3.70
Mean ± SD	1495.4 ± 68.12	56.3 ± 3.33**	3.76 ± 0.08**
H1	1701.5	133.8	7.86
H2	1750.1	135.8	7.76
H3	1596.9	122.6	7.68
Mean ± SD	1682.83 ± 45.20	130.73 ± 4.11	7.77 ± 0.05

### MeRIP-seq and mRNA-seq

The 6 adipose tissues were divided into high-fat (HF) and low-fat (LF) groups based on their abdominal fat ratio. Total RNA was extracted from the fat tissues and DNA was removed using Dnase I. Then the rRNA was removed. The RNA was then chopped into small fragments, added to antibodies for binding and enriched for m^6^A. The IP library and input library were constructed and MeRIP-seq and mRNA-seq were performed using the Hiseq platform, which was then complemented by E-GENE technology.

### Cell culture and cell transfection

Immortalised chicken preadipocyte 1 (ICP-1) was provided by the Li Hui laboratory of Northeast Agricultural University (Heilongjiang, China) [37, 38]. ICP-1 was cultured in a mixed medium of DMEM and DMEM/ F_12_ (Gibco, CA, United States) with 15% fetal bovine serum (Gibco, CA, United States) and 1% streptomycin/penicillin (Invitrogen, CA, United States) at 37 °C with 5% CO_2_. Lipofectamine 3000 (Invitrogen, CA, United States) was used for cell transfection. According to the manufacturer’s instructions, the transfection dose of plasmids or oligonucleotides for different plates was as follows 14–28 µg for each 10 cm plate, and 1 µg/well or 50 nM/well for 12-well plates.

### Total RNA extraction

Total RNA extraction from cells and tissues was performed using TriQuick Reagent (SolarBio, Beijing, China) according to manufacturer’s instructions. A total of 50 mg of homogenised tissue or 10^6^ cells were lysed by adding 1 mL of TriQuick Reagent (SolarBio, Beijing, China) at room temperature. 200 µL chloroform (Damao, Tianjin, China) was added, mixed, allowed to stand at room temperature for 2–3 min and centrifuged at 12,000 × g at 4 ℃ for 15 min. The upper aqueous phase was transferred to a new 1.5 mL centrifuge tube. Next, 500 µL isopropanol (Damao, Tianjin, China) was added to the tube, left at room temperature for 10 min to precipitate the RNA, centrifuged at 12,000 × g at 4 ℃ for 10 min, the supernatant was discarded, and 1 mL 75% ethanol (Damao, Tianjin, China). The mixture was centrifuged at 12,000×g at 4 ℃ for 10 min and the supernatant was discarded. Then, 1 mL of 75% ethanol was added to the tube (Damao, Tianjin, China) and centrifuged at 12,000 × g at 4 ℃ for 2 min. Finally, 25 µL of RNase-free water was added to the tubes to the dissolve RNA (SolarBio, Beijing, China), and stored at -80℃.

### Plasmids construction and oligonucleotides synthesis

The complete CDS sequence of *ALKBH5* (XM_004945197.4) was synthesised by TSINGKE (Beijing, China) and subcloned into the Nhe I and Hind III sites of the pcDNA3.1-Flag vector (Promega, WI, United States). And the complete CDS sequence of *LCAT* (NM_001293094.2) was subcloned into the Hind III and BamH I sites of pcDNA3.1 vector (Promega, WI, United States) by TSINGKE (Beijing, China). The plasmids were named as *ALKBH5* and *LCAT*-pcDNA3.1. In addition, siRNA of *LCAT* was synthesized by RioBio (Guangdong, China), and the sequence of the oligonucleotides is listed in Table 2.

**Table 2:** The sequence information of oligonucleotide

Fragment name	Fragment sequences (5’ to 3’)
si-*LCAT*	CCTGGCGTCAGGTGATAAT

### Flow cytometry analysis

After 48 h of transfection, the cells were digested with 0.25%-EDTA trypsin (Gibco, CA, United States). After washing with PBS, the cells were immobilised in 75% pre-cooled ethanol. The fixative was removed, and the cells were washed with PBS. Finally, 0.5 mL of propidium iodide (PI) solution (Beyotime, Shanghai, China) was added and the cell pellet was gently resuspended. The cells were incubated at 37℃ for 30 min without light. Cells were analysed using a CytoFlex instrument (Beckman, CA, United States).

### Oil red O staining

After 6 h of transfection, preadipocyte differentiation was induced using a medium containing 15% fetal bovine serum and 0.2% oleic acid (Sigma, CA, United States). After 48 h of induction, the cells were washed with PBS and fixed with 300 µL of 4% paraformaldehyde for 30 min at room temperature. The cells were washed twice with PBS, 300 µL of Oil Red O Staining Solution (SolarBio, Beijing, China) was added and incubated at room temperature for 1 h. The staining solution was then removed and the cells were washed four times with PBS. The stained cells were observed and photographed under an electron microscope (Nikon, Tokyo, Japan). Finally, 300 µL of isopropyl alcohol was used to clean the stained cells and absorbance values were determined at 510 nm (bio-rad, CA, United States).

### MeRIP-qPCR

The Magna MeRIP™ m^6^A (MeRIP) Kit (Magna, United States) was used to detect m^6^A methylation in cells according to the manufacturer’s protocol. 300 µg of RNA was fragmented using RNA Fragmentation Buffer at 94 °C for 5 min. End fragmentation with the addition of 0.5 M EDTA. Then 3 M sodium acetate, glycogen (100 µg/mL) and anhydrous ethanol were added, mixed and incubated at -80 ℃ overnight. The samples were then centrifuged at 15,000 × g for 25 min at 4 ℃, the supernatant was discarded, 1 mL of 75% ethanol was added and centrifuged at 15,000 × g for 15 min at 4 ℃. Finally add 300 µL of RNase-free water and store at -80 °C. Take 25 µL of Magnetic Beads A/G Blend and add 10 µL to each tube. m^6^A antibody or normal mouse IgG antibody was incubated for 30 min at room temperature, adsorbed on a magnetic rack and the supernatant was discarded. 10 µL of fragmented RNA was taken as input, the remaining RNA was added to RNase inhibitor and IP buffer 5×, and finally RNASe-free water was added to l mL. 500 µL was added to the immunoprecipitation-loaded magnetic beads, incubated at 4 ℃ for 2 h, washed twice with IP buffer, eluent added to each tube, and eluted twice at 4 ℃ for 1 h each time. Finally, RNA was purified and the purified RNA was used for reverse transcription and RT-qPCR.

### m^6^A dot blot

The m^6^A Dot Blot was used to detect the total RNA methylation level in the cells. After 48 h of transfection, total RNA from ICP-1 cells was harvested and 1 µg RNA was denatured at 95 ℃ for 3 min. The denatured RNA was cross-linked to a positively and negatively charged nylon membrane. The unbound RNA was then washed with TBST for 5 min. After blocking with 5% skimmed milk for 1 h, the membrane was incubated with anti-m^6^A (1 : 500; ab151230, abcam), and anti-mouse IgG antibodies (1 : 5,000; ab190475, abcam) at 4 °C overnight. The membrane is not cut prior to hybridisation with the antibody. Finally, Odyssey Instrument (Li-cor, CA, United States) and ImageJ Software were used for visual analysis and grey scale analysis of the membrane.

### RNA binding protein immunoprecipitation (RIP)

After 48 h of transfection, the RNA-Binding Protein Immunoprecipitation kit (Magan, United States) was used to detect the binding between *LCAT* mRNA and *ALKBH5* in cells. The RIP assay was performed according to the manufacturer’s protocol for RNA-Binding Protein Immunoprecipitation (Magan, United States).

### Reverse transcription and real-time quantitative PCR (qPCR)

Reverse transcription PCR for mRNA was performed using MonScriptTM RTlll All-in-one Mix (Monad, Wuhan, China) according to the manufacturer’s protocol. ChamQ Universal SYBR qPCR Master Mix (Vazyme, Nanjing, China) and ABI QuantStudio 5 (Thermo Fisher, NY, United States) were used for real-time fluorescence quantification. NCBI Primer Design was used to design qPCR primers, and primer information is provided in Supplementary file 1 Table S1. The mRNA of *GAPDH* was used as an internal control.

### Prediction of methylation sites of m^6^A

According to the methylation modification sites given by the sequencing results, the m^6^A methylation sites of *LCAT* were predicted using http://genome.ucsc.edu/. The sequence of the predicted sites is shown in Supplementary file 1 Table S2. According to the predicted results, the primers for qPCR were designed using Premier Primer 5.0 software and the primer sequences are shown in Supplementary file 1 Table S3.

### Actinomycin D treatment

*ALKBH5* was overexpressed in ICP cells and treated with Actinomycin D 24 h later. 5 µg/well Actinomycin D was added to a 12-well plate. Cells were harvested at 0 h, 3 h and 6 h and *LCAT* expression levels were determined at different time points.

### Western blotting

Proteins were extracted from cells after 48 h of transfection using ice-cold radio immunoprecipitation (RIPA) lysis buffer containing 1 mM phenylmethyl sulfonyl fluoride (Beyotime, Shanghai, China). Protein samples were separated on a 12% SDS-PAGE gel (EpiZyme, Shanghai, China) run at 120 V for 60 min. The proteins were transferred to polyvinylidene fluoride (PVDF) membrane (Bio-Rad, CA, United States). After blocking with 5% skim milk for 1 h and the membrane is sheared at 38 kDa. The membrane was incubated with anti-PPARγ (1 : 1,000; bs-0530R, BIOSS), ALKBH5 (1 : 1,000; 49,015, CST), LCAT (1 : 1,000; CQA2612; CohesionBio) and anti-GAPDH (1 : 2,000; bsm-33,033 M, BIOSS) antibodies at 4 °C overnight. Anti-rabbit IgG HRP-antibody (1 : 10,000; 7074P2, CST) was used as secondary antibody. Finally, the protein bands were visualised using Odyssey (Li-COR, CA, USA) and the grey value (image density) was measured using ImageJ software. Alternatively, the film can be exposed by inserting it into an X-ray clamp.

### Data statistics and analysis

Student’s t-test was used to compare the differences between groups. Each group had at least three between-group replicates. All experimental data are expressed as mean ± SEM. ∗*P* < 0.05; ∗∗*P* < 0.01; ns: no significant.

## Results

### MeRIP-seq analysis of m^6^A methylation in High-Fat Group and Low-Fat Group

**Fig. 1:**
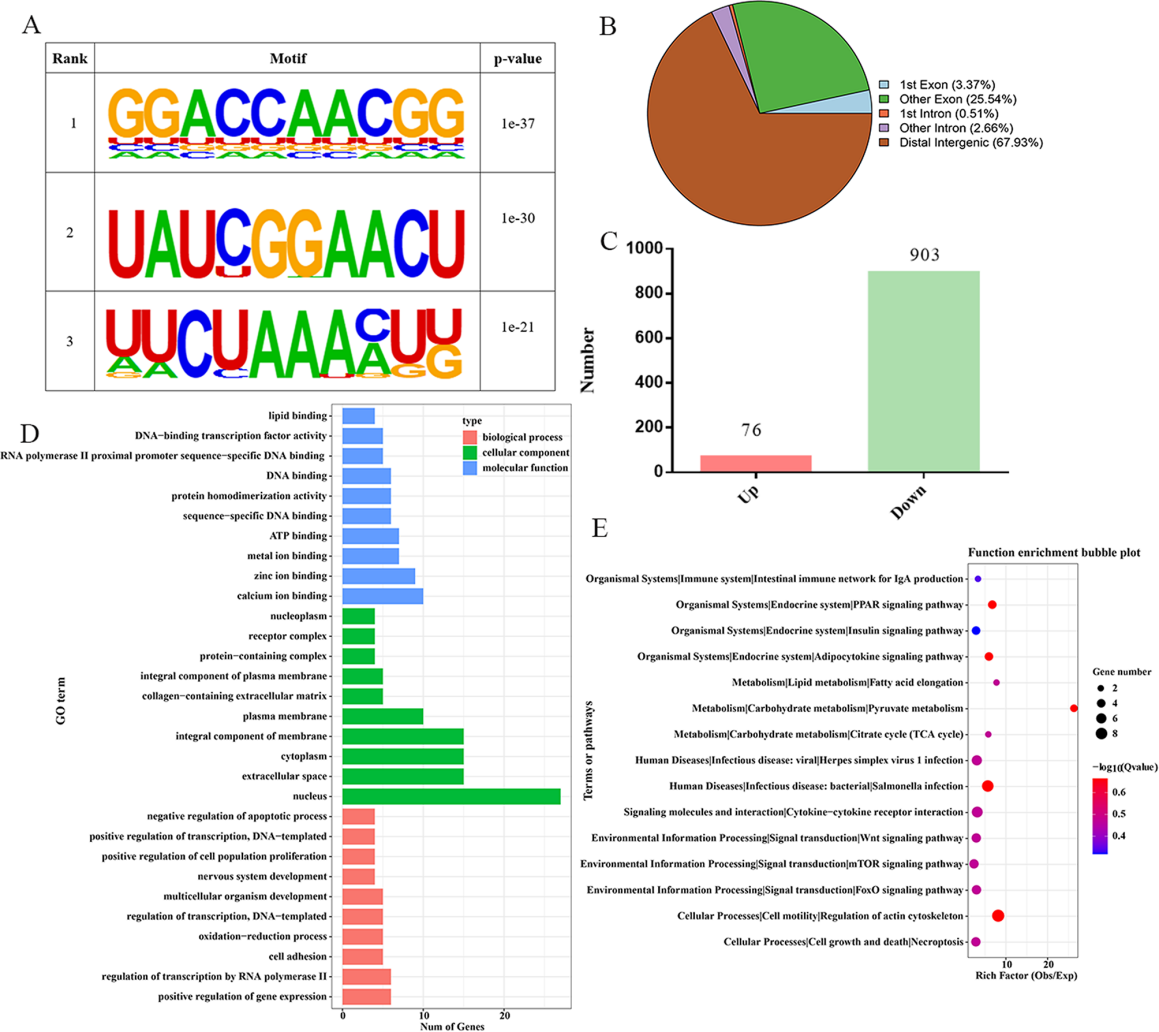
Analysis of MeRIP-seq in chicken abdominal fat tissues between he high-fat and low-fat groups. **(A)** The regional distribution of difference m^6^A methylation modification enrichment. **(B)** Markedly different motif types. **(C)** Statistical analysis of the number of different m^6^A modification sites in the high-fat group and the low-fat group. **(D)** Genes associated with difference m^6^A methylation modification sites enriched GO term. X axis: gene number; Y axis: GO terms. **(E)** Genes associated with difference m^6^A methylation modification sites enriched KEGG pathways. X axis: rich factor; Y axis: pathway

From an epigenetic perspective, the aim of this study was to investigate the regulatory mechanism of RNA methylation in chicken fat deposition. Abdominal adipose tissues from 3 high-fat (HF) and 3 low-fat (LF) broilers were selected for MeRIP-seq. Quality control results indicated that the percentage of Q30 (base detection accuracy ≥ 99.9%) was greater than 97% (Supplementary file 1 Table S4). In addition, at least 17,008 peaks were obtained for each sample (Supplementary file 1 Table S5). All raw data were submitted to the NCBI SRA database. |Fold Change| ≥ 2, and P-value<0.05 were set as differential screening criteria, a total of 979 differential m^6^A methylation modification sites were obtained by performing MeRIP-seq in abdominal fat RNA. The most common motif type was RRACH, and the three motifs with the most significant differences were GGACA, GAACU and AAACU (Fig. 1A). The distribution of peaks in gene elements with significant differences was statistically analysed and it was found that most of the peaks were located in distal intergenic regions (Fig. 1B). Among the differential m^6^A methylation modification sites, 76 were up-regulated and 903 were down-regulated (Fig. 1C). The results of GO function analysis of genes associated with differential m^6^A methylation modification sites showed that these genes could be enriched in cell differentiation, nucleus and ATP binding, glucose transmembrane transport, growth and glucose transmembrane transport activity (Fig. 1D). KEGG pathway enrichment analysis showed that these genes may be enriched in the PPAR signalling pathway, adipokine signalling pathway, fatty acid elongation and FOXO signalling pathway, suggesting that these genes may be involved in chicken adipogenesis (Fig. 1E). The top 20 abundant m^6^A methylation modification sites are listed in Table 3, including *LOC396477*, *C11H16ORF87*, and *CDCA9*. Through GO and KEGG analysis, some differential m^6^A methylation modifier genes (*LCAT*, *PDK4*, *PRMT7*, *SIRT6*, *SLC2A1* and *SIRT5*), which may be related to lipid metabolism, were considered as potential candidates in the subsequent study [39–41].

**Table 3:** The top 20 abundant mRNA with differences in m^6^A methylation modification sites

Chrom	PeakStart	PeakEnd	mRNA	Foldchange	Regulation
chr30	1500775	1504141	*LOC396477*	429	Up
chr11	7757976	7758485	*C11H16ORF87*	66.6	Up
chr11	7757976	7766226	*C11H16ORF87*	65.1	Up
chr11	7757926	7758496	*CDCA9*	56.3	Up
chr7	12131545	12132291	*FZD5*	45	Up
chr25	3853425	3854692	*CRP*	43.3	Up
chr19	7780459	7782595	*TBX4*	39	Up
chr23	2823327	2824045	*SRRM1*	34.2	Up
chr5	38323050	38324277	*ACYP1*	212	Down
chrZ	7906219	7906869	*ENHO*	148	Down
chr1	1.96E + 08	1.96E + 08	*ART7B*	103	Down
chr2	1.21E + 08	1.21E + 08	*STMN2*	82.5	Down
chr13	2400602	2401232	*PCDHGC3*	78.1	Down
chr19	3576183	3577516	*P2RX1*	77.4	Down
chr9	3926622	3927368	*CEP63*	75.9	Down
chr7	28520894	28521342	*C7H2ORF76*	57.7	Down
chr27	7209722	7210406	*RARA*	55.8	Down
chr18	806620	807041	*MYOCD*	55.8	Down
chr30	389347	389704	*HUC*	55.8	Down
chr23	5630172	5631159	*PPT1*	35.2	Down
chr13	11172623	11175401	*ARL10*	32.5	Down

### Genome-wide mRNA screening and analysis identified mRNA Associated with Fat Deposition

**Fig. 2:**
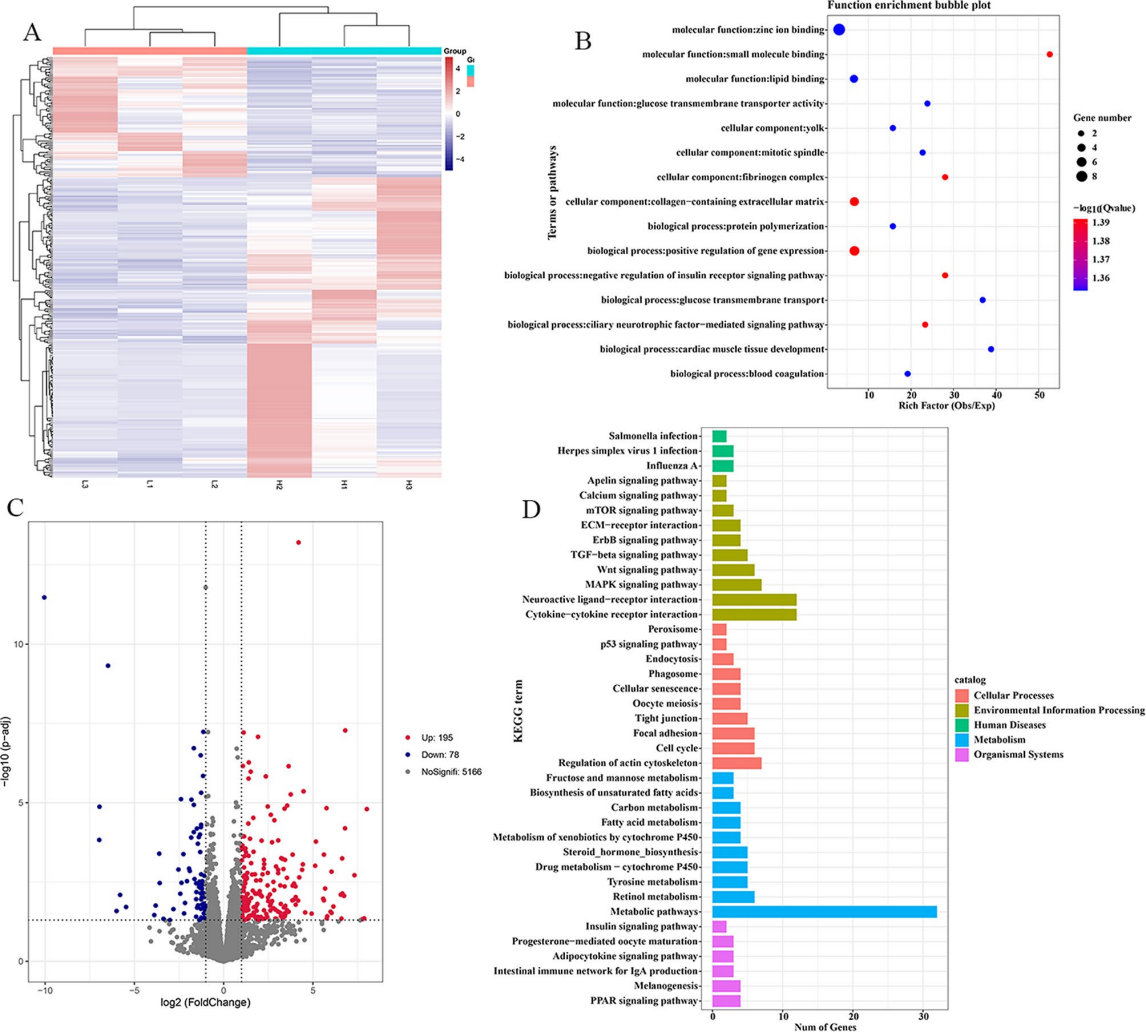
Analysis of mRNA differentially expressed in chicken abdominal fat tissues between the high-fat and low-fat groups. (**A**, **C**) Heat map and Volcano plot for low-fat_VS_high-fat DEGs. (**B**) The GO function enrichment analysis for DEGs. (**D**) The KEGG pathways classification analysis for DEGs.

To explore the regulatory mechanism of abdominal fat deposition in chickens, mRNA sequencing was performed on the same samples and submitted to the NCBI SRA database. For RNA-seq, |Fold Change| ≥ 2, and P-value<0.05 were considered as screening criteria for differentially expressed genes (DEGs). A total of 273 DEGs were obtained (Fig. 2A). Among them, 195 genes were up-regulated and 78 genes were down-regulated in the high-fat group (Fig. 2C). GO functional analysis showed that these DEGs could be enriched in glucose transmembrane transport, glucose transmembrane transporter activity, fatty acid metabolism, and lipid binding (Fig. 2B, D). Some genes related to fat deposition were identified by GO and KEGG analysis, suggesting that these genes might be involved in abdominal adipogenesis in chickens, including *SLC2A2*, *PMP2*, *SLC2A1*, *HNF4A*, *FABP7*, *PDK4* and *LCAT* (Table 4) [42–44].

**Table 4:** Differentially expressed mrnas associated with fat formation and lipid metabolism

Gene name	Low-Fat Expression	High-Fat Expression	log2Ratio (HF/LF)	*P* value	Up Down	Gene description
*HNF4A*	0	47.98	8.020987199	1.58E-05	Up	hepatocyte nuclear factor 4 alpha
*SLC2A2*	4.35	133.34	4.93083747	0.031024063	Up	facilitated glucose transporter
*PMP2*	9.9	116.91	3.558754643	1.23E-05	Up	fatty acid binding protein 9
*FABP1*	15.18	137.28	3.179519135	0.003994144	Up	Fat body protein 1
*ELOVL6*	223.13	973.74	2.125485279	0.00015671	Up	ELOVL fatty acid elongase 6
*FABP7*	115.85	348.89	1.590417855	0.001824541	Up	fatty acid binding protein 7
*INSIG1*	973.75	1982.43	1.025331849	0.000645623	Up	insulin induced gene 1
*SLC2A1*	1105.01	504.49	-1.131247985	5.76E-08	Down	facilitated glucose transporter
*PDK4*	15911.74	7564.62	-1.07276	0.022237047	Down	pyruvate dehydrogenase kinase 4
*LCAT*	273.63	717.75	1.390884877	0.044774122	Up	lecithin-cholesterol acyltransferase

### **Combined analysis of MeRIP-seq and mRNA-seq Indicated that mRNA methylation of ***LCAT ***may regulate fat deposition**

**Fig. 3:**
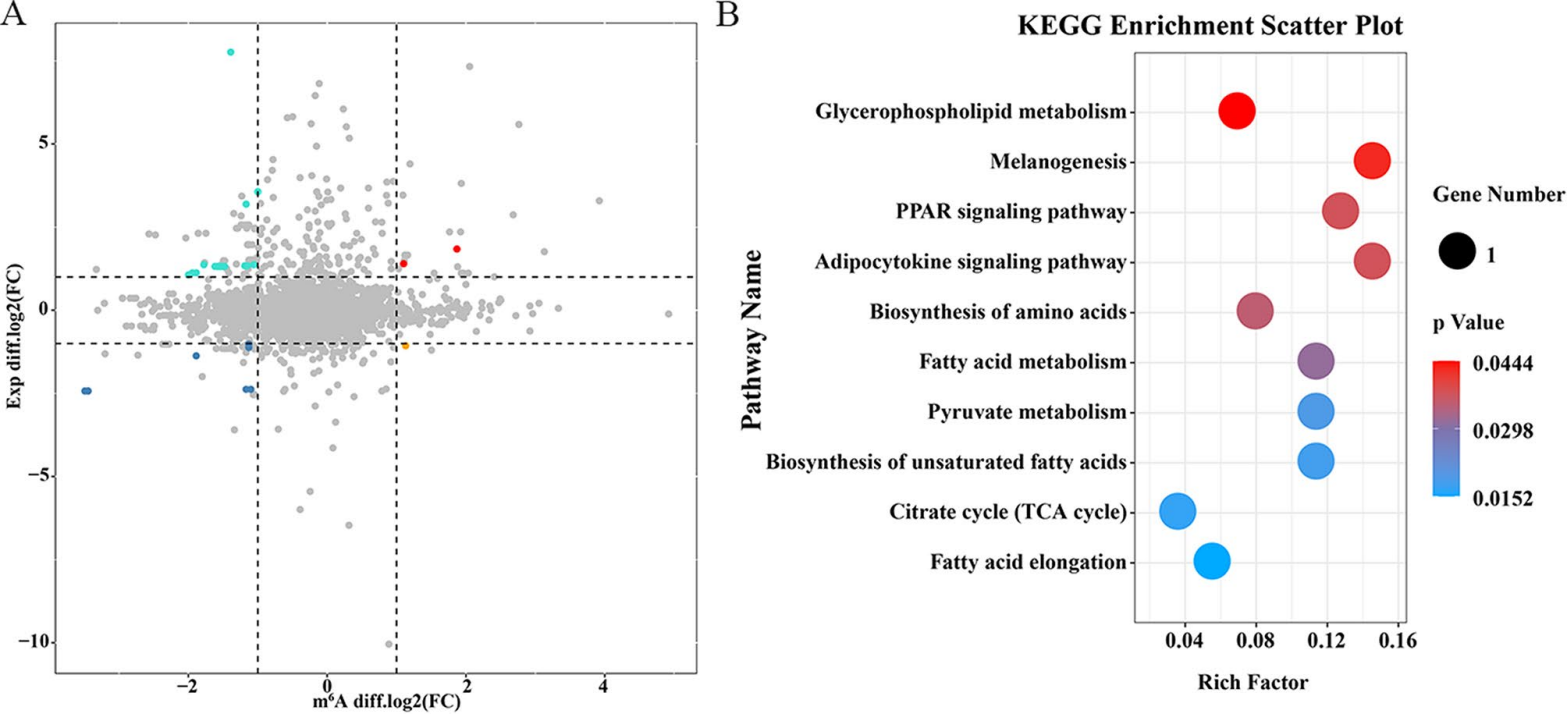
Combined analysis of MeRIP-seq and mRNA-seq. **(A)** Four quadrants between differential m^6^A methylation modification associated genes and differential expression genes. **(B)** The KEGG pathways enrichment analysis for differential m^6^A methylation modification associated genes and differential expression genes

To further understand the relationship between m^6^A methylation modification and mRNA expression in abdominal fat deposition, we performed association analysis on the omics data of MeRIP-seq and mRNA-seq. A total of 16 DEGs were found to be associated with differential m^6^A methylation modifications, suggesting that these m^6^A methylation modifications may regulate their mRNA expression (Fig. 3A). KEGG enrichment analysis of these 16 genes showed that these genes were most significantly enriched in the lipid metabolism pathway (Fig. 3B), among which the adipose-related genes were *ELOVL2*, *PDK4*, *PMP2*, *FABP1*, *LAMP3*, *LCAT* and *SLC2A1* (Table 5) [45–47]. Combined with MeRIP-seq and mRNA-seq, it was found that *LCAT* mRNA level was positively correlated with the reduction of its mRNA methylation level, indicating that *LCAT* mRNA stability may be responsible for adipogenesis in chickens.

**Table 5:** Differential m^6^A modification and DEGs associated with lipid metabolism

Chrom	PeakStart	PeakEnd	mRNA	Foldchange	Regulation
chr2	62916247	62916723	*ELOVL2*	10.7	Down
chr2	23907156	23907546	*PDK4*	7.74	Up
chr2	1.21E + 08	1.21E + 08	*PMP2*	27.9	Down
chr4	86132634	86135786	*FABP1*	2.72	Down
chr9	16523508	16524286	*LAMP3*	10.5	Down
chr11	1035168	1036088	*LCAT*	12.5	Up
chr21	6462015	6462525	*SLC2A1*	10.7	Down
chr33	6174474	6190421	*PC*	6.37	Down

### **ALKBH5, as a demethylase, mediated the degradation of ***LCAT ***and affect its stability**

**Fig. 4:**
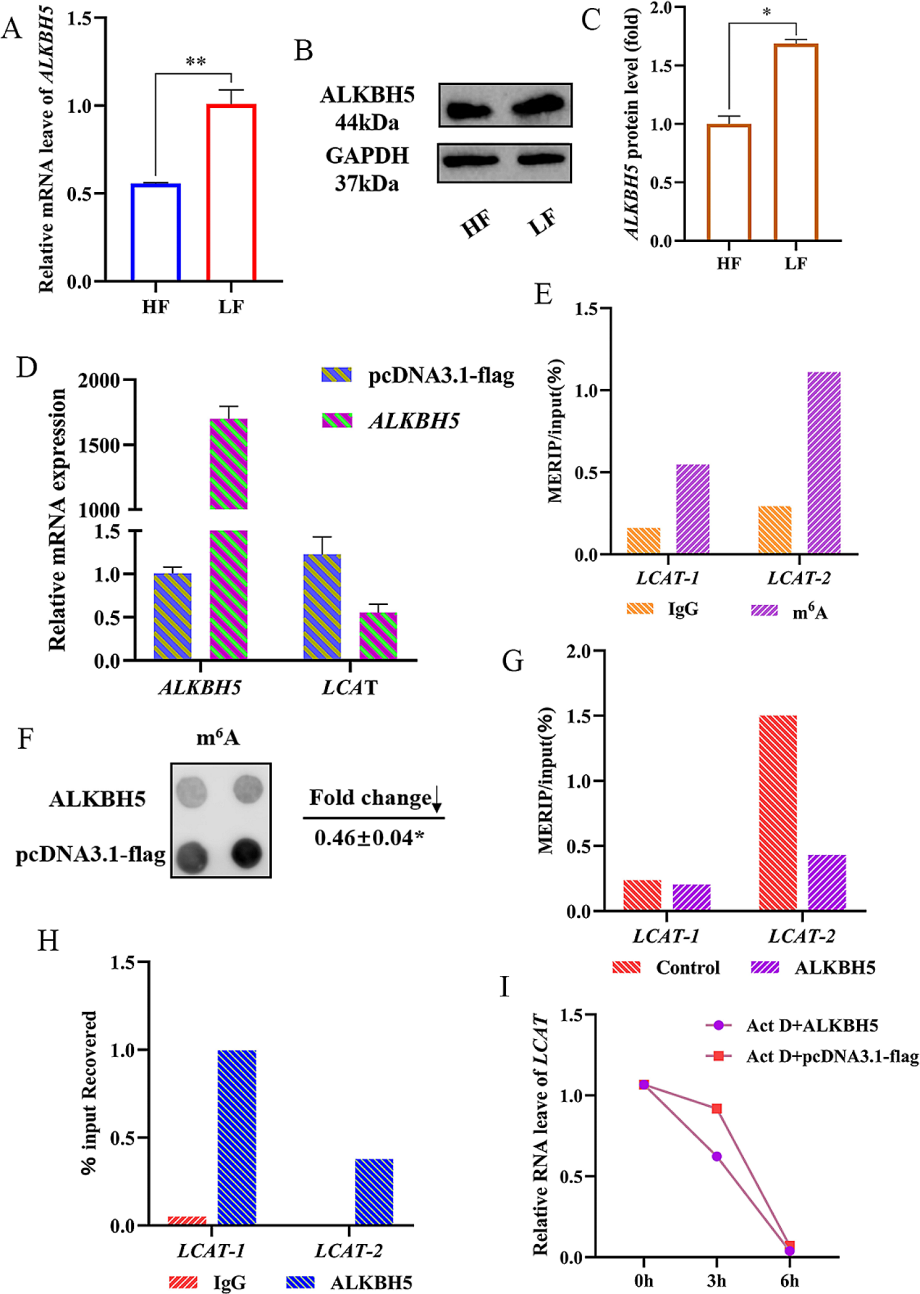
ALKBH5 mediates LCAT stabilization through demethylation. **(A)** Relative expression of *ALKBH5* in high-fat chicken and low-fat chicken. (**B**, **C**) The western blot for *ALKBH5* protein in HF and LF, full-length blots are presented in Supplementary file 3 B. **(D)** Transfection efficiency of *ALKBH5* and relative expression of *LCAT*. **(E)** The methylation modification sites of LCAT were detected by MeRIP-qPCR. **(F)** m^6^A Dot Blot was used to detect the overall RNA methylation of ICP-1 cells after transfection with *ALKBH5*, full-length blots are presented in Supplementary file 3 A. **(G)** Methylation of *LCAT* mRNA after *ALKBH5* overexpression was detected by MeRIP-qPCR. (H) ALKBH5 protein was detected to bind to RNA of *LCAT* by RIP. **(I)** After transfection of *ALKBH5*, cells were treated with 5 µg/mL Actinomycin D to detect the relative expression of *LCAT*. Data presented as mean ± SEM, *P < 0.05; **P < 0.01;ns: no significance

*ALKBH5* is differentially expressed in chicken abdominal adipose tissue between HF and LF groups, prompting an investigation into its role in demethylation and its functional impact on chicken abdominal adipose tissue(Fig. 4A, B and C). We constructed an *ALKBH5* overexpression vector and qPCR results showed that *LCAT* expression was significantly downregulated when *ALKBH5* was overexpressed (Fig. 4D). This suggests that *ALKBH5* affects the expression of *LCAT*. This was followed by MeRIP-qPCR to confirm that the m^6^A sites were present on *LCAT* mRNA. Compared to the IgG group, IP production using the m^6^A specific antibody showed an enrichment of *LCAT* mRNA, which was consistent with the sequencing results (Fig. 4E). To further investigate the effect of *ALKBH5* on *LCAT* of preadipocytes, we overexpressed *ALKBH5* on ICP, and Dot Blot showed that ALKBH5 down-regulated the total RNA methylation level of preadipocytes compared to the blank group (Fig. 4F). Meanwhile, MeRIP-qPCR was used to confirm *ALKBH5* mediated *LCAT* mRNA demethylation upon *ALKBH5* overexpression, and *LCAT* mRNA enrichment was reduced in the *ALKBH5* group compared to the control (Fig. 4G). RIP was used to further investigate whether LCAT binds AKLBH5, and the results showed that *LCAT* mRNA was enriched in the ALKBH5 group compared to the IgG group. AKLBH5 protein was shown to be bound to *LCAT* mRNA (Fig. 4H). In addition, ALKBH5 overexpressing preadipocytes were treated with ActinomycinD, and qPCR results showed that ALKBH5 continuously down-regulated the expression of *LCAT* at different treatment times compared with the blank group. At the same time, *ALKBH5* down-regulated the expression of *LCAT* at the same treatment time (Fig. 4I). In conclusion, the Dot Blot results demonstrated that *ALKBH5* could down-regulate the methylation level of ICP cells in a global manner. Subsequently, MeRIP-qPCR and RIP experiments demonstrated that *ALKBH5* binds to the methylation modification site on *LCAT* mRNA, and that *ALKBH5* further influenced the stability of *LCAT* following treatment with actinomycin D.

### *LCAT***inhibits the proliferation of preadipocyte**

**Fig. 5:**
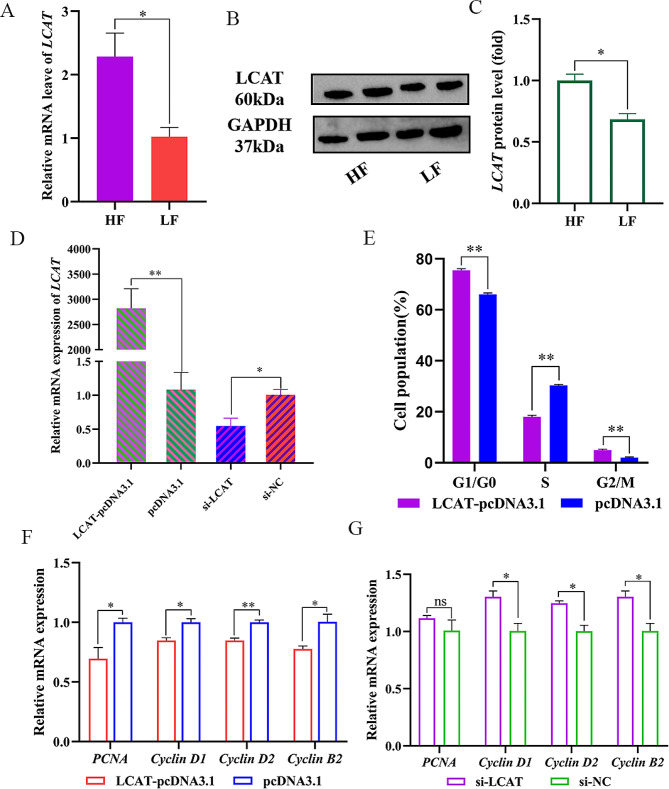
*LCAT* inhibits the proliferation of preadipocyte. **(A)** Relative expression of LCAT in high-fat chicken and low-fat chicken. (**B**, **C**) The western blot for *LCAT* protein in HF and LF, full-length blots are presented in Supplementary file 3 C. **(D)** Transfection efficiency of *LCAT* and si-*LCAT*. **(E)** Flow cytometry for cell cycle detection of ICP-1 cells. (**F**, **G**) Relative mRNA expression of PCNA, Cyclin D1, ,Cyclin D2, and Cyclin B2 in ICP-1 cells. Data presented as mean ± SEM, *P < 0.05; **P < 0.01;ns: no significance

The qPCR and western blot results showed that *LCAT* was differentially expressed in adipose tissue between HF and LF, which was consistent with the sequencing data (Fig. 5A, B and C). To further explore the role of *LCAT* during fat deposition in chickens, we continued the study of cellular function. We constructed the plasmid and siRNA to overexpress or knock down *LCAT* (Fig. 5D). Flow cytometry results showed that the preadipocyte cycle stagnated in G1 phase after *LCAT* overexpression, leading to a decreased proportion of S phase cells (Fig. 5E and Supplementary file 2 Fig S1). Meanwhile, the qPCR results showed that *LCAT* overexpression up-regulated the expression of *PCNA*, *Cyclin D1*, *Cyclin D2* and *Cyclin B2* (Fig. 5F). On the contrary, *LCAT* knockdown promoted their expression (Fig. 5G). It is speculated that *LCAT* suppresses preadipocyte proliferation.

### *LCAT***Promotes differentiation of preadipocyte through PPARγ pathway**

**Fig. 6:**
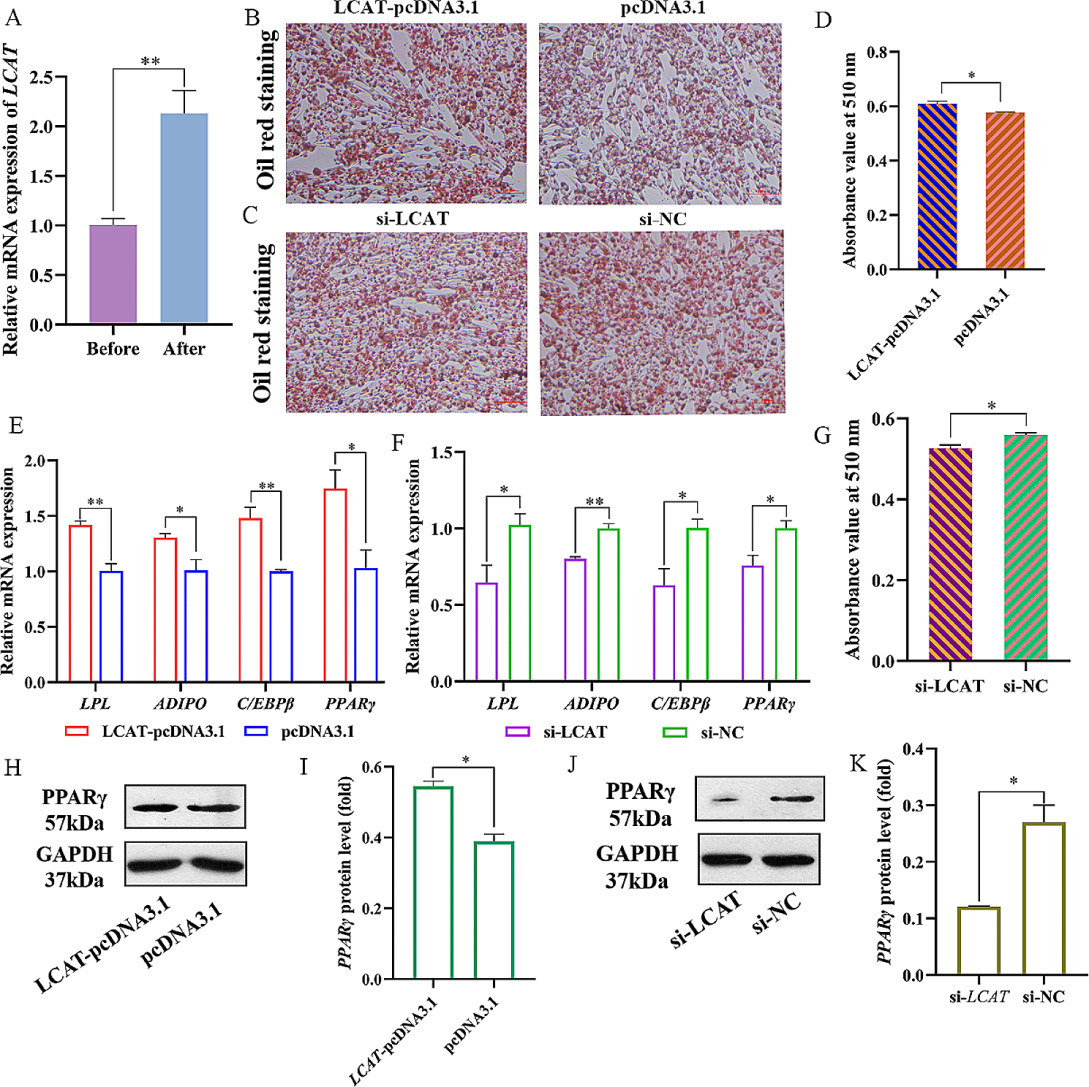
*LCAT* promotes preadipocytes differentiation via PPARγ pathway. **(A)** Relative expression of *LCAT* before and after preadipocytes differentiation. (**B**, **C**) Oil red staining for lipid droplet formation. (**D**, **G**) Lipid droplets change (**E**, **F**) Relative mRNA expression of *LPL*, *ADIPO*, *C/EBPβ*, and *PPARγ* in ICP-1 cells. (**H** - **K**) The western blot for *PPARγ* protein in ICP-1 cells, full-length blots are presented in Supplementary file 3 D. Data presented as mean ± SEM, *P < 0.05; **P < 0.01; ns: no significance

We further explored the role of *LCAT* in preadipocyte differentiation. First, oleic acid was used to induce preadipocyte differentiation. The results of qPCR showed that *LCAT* expression increased significantly after differentiation induction (Fig. 6A). Meanwhile, Oil Red O staining showed that *LCAT* promoted the formation of lipid droplets during the differentiation of preadipocytes compared with the blank group (Fig. 6B, D). In contrast, *LCAT* knockdown inhibited lipid droplet formation (Fig. 6C, G). In addition, *LCAT* overexpression promoted the mRNA expression of *LPL* and *PPARγ*, which are considered to be the key preadipocyte differentiation genes in the PPARγ pathway. It also promoted the expression of *ADIPO* and *C/EBPβ* (Fig. 6E, F). The protein levels of PPARγ were increased after *LCAT* overexpression in preadipocytes, whereas *LCTA* knockdown decreased the protein expression (Fig. 6H - K). Taken together, these results suggest that *LCAT* overexpression may accelerate preadipocyte differentiation by promoting the activation of the PPARγ pathway.

## Discussion

In poultry, abdominal adipogenesis causes a number of metabolic diseases and feed wastage. Recent studies have shown that m^6^A methylation is involved in the regulation of adipogenesis and plays an important role in the process of adipogenic differentiation [48, 49]. Unlike previous studies focusing on m^6^A methylation in mammalian systems, our research provides the first evidence of m^6^A’s role in regulating *LCAT* mRNA stability and adipogenesis in chickens. Our findings suggest that m^6^A methylation is a critical regulatory mechanism in avian species, with potential implications for understanding and manipulating fat metabolism across different organisms. In this study, we used the sequencing technology to identify a differential gene (*LCAT*) and its mRNA with potential m^6^A modification sites that were significantly associated with abdominal fat deposition. As an m^6^A demethylase, ALKBH5 is involved in a variety of biological regulation and controls RNA fate in an m^6^A dependent manner [50, 51]. In the previous experiment, we found that ALKBH5 was highly expressed in the low-fat group. Based on the sequencing data, we designed a series of molecular and cellular experiments to explore the demethylation of ALKBH5 on LCAT and the role of *LCAT* in fat deposition. Our results showed that ALKBH5 could regulate the RNA methylation of *LCAT*, affect the stability and promote *LCAT* mRNA. We also found that *LCAT* inhibited the proliferation and promoted differentiation of preadipocytes.

Various transcripts, such as mRNA, lncRNA, and circRNA, have m^6^A methylation modification, which can affect the stability and post-transcriptional processing of RNA [52, 53]. The m^6^A methylation sites in the coding sequence regions (CDS) play an important role in the stability of mRNA [54]. Zhao et al. found that FTO controls the exon splicing of the lipid-forming regulator RUNX1T1 by regulating the level of m^6^a modification around the splicing site in the CDS regions, to regulate the differentiation [55]. The study by Zhang et al. shows that *METTL3* can regulate mRNA translation through methylation of the CDs region of *LDHA* [56]. Our sequencing data showed that the m^6^A methylation modification site of *LCAT* was located in the CDS region, and the degree of m^6^A methylation modification was greater in the high-fat group than that in the low-fat group. To determine whether *ALKBH5* regulates *LCAT* expression through means of m^6^A dependence. The qPCR, MeRIP-qPCR, RIP and Actinomycin D treatments were used in the chicken preadipocyte model. The results showed that the demethylation of *ALKBH5* accelerated the degradation of *LCAT* mRNA, indicating that the mRNA methylation in the CDS region of *LCAT* enhanced its stability. Studies have shown that m^6^A’s regulatory factors are linked to fat [36, 57]. Overexpression of *ALKBH5* increases *FABP5* expression in a m^6^A-*IGF2BP2* dependent manner, leading to lipid metabolism disorders [58].The research report that inhibition of *ALKBH5* could promote m^6^A modification of *CCL28* mRNA and increase its stability [59]. The regulatory mechanism is the same as for this experiment.

*LCAT* has been extensively studied in atherosclerosis over the last few decades [22, 25, 60]. However, little is known about its role in fat deposition. Studies have shown that *LCAT* deficiency provides specific protection against diet-induced obesity and insulin resistance by regulating white adipose tissue formation and brown fat cell allocation [61]. Based on the evidence that *LCAT* may be involved in adipose tissue deposition, we further investigated its role in this biological function. Xepapadaki reported that loss of *APOAL* or *LCAT* in mice led to increased sensitivity to body weight, and *LCAT* deficiency resulted in a synergistic reduction in oxidative phosphorylation and non-shivering thermoproduction in white adipose tissue [62]. The expression of *LCAT* has been found to be up-regulated in the treatment of obese people [63]. These data are consistent with our results. In this experiment, *LCAT* expression level was less in the high-fat group. In chicken proadipocytes, flow cytometry showed that *LCAT* inhibited the G1 to S phase transition, and qPCR showed that *LCAT* inhibited the expression of cell cycli-related genes. Meanwhile, *LCAT* promoted the PPARγ signalling pathway at the protein and RNA levels, and the effects of *LCAT* overexpression and interference on preadipocyte differentiation were determined by qPCR and Oil red O staining. However, the research by Nesan et al. showed that *LCAT* deficiency could promote the differentiation of cholesterol-dependent satellite cells into brown adipocytes [24]. This is contrary to our results, and the reasons for this difference are unclear. Due to the limited size of the sequencing sample employed in this experiment, it is possible that certain limitations may be encountered. In order to validate our findings and improve the generality of the results, larger and more diverse studies will be conducted in the future.

## Conclusions

**Fig. 7:**
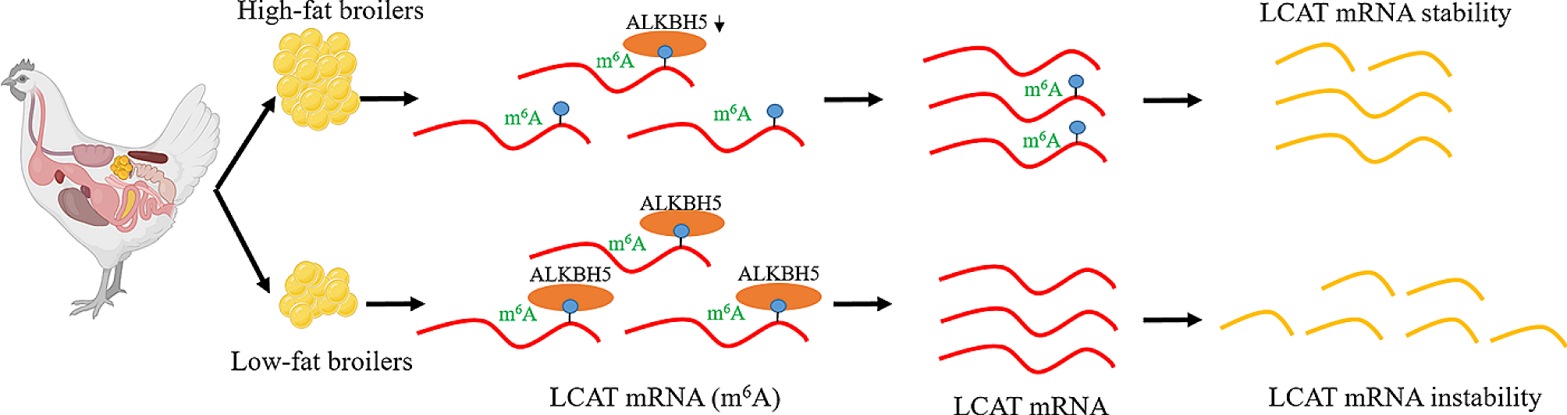
Schematic diagram of the role of RNA methylation modification of *LCAT* mRNA in abdominal fat deposition in chickens.*ALKBH5* affects the stability of *LCAT* mRNA through demethylation modification, and *LCAT* affects the proliferation and differentiation of ICP cells

In summary, our results indicate that *LCAT* expression and the degree of m^6^A methylation modification were down-regulated in the low-fat group. In addition, we demonstrated the relationship between *ALKBH5* and *LCAT*, and confirmed that *ALKBH5* affects the stability of *LCAT* mRNA through demethylation (Fig. 7). Whereas *LCAT* inhibits preadipocyte formation and promotes differentiation. This study identified, *ALKBH5* was identified to be involved in adipogenesis by demethylation mediating *LCAT* mRNA stability. It is expected to provide evidence for the role of RNA methylation in fat process.

### Electronic supplementary material

Below is the link to the electronic supplementary material.

Supplementary Material 1

Supplementary Material 2

Supplementary Material 3

## Data Availability

Sequence data that support the findings of this study have been deposited in the NCBI database of SRA.The accession number of RNA methylation data is PRJNA876894, accession link1: http://www.ncbi.nlm.nih.gov/bioproject/876894, and the accession number of mRNA data is PRJNA876840, accession link2: http://www.ncbi.nlm.nih.gov/bioproject/876840.

## Abbreviations

m^6^A: N6-methyloint
ALKBH5: AlkB Homolog 5, RNA Demethylase
LCAT: Lecithin-cholesterol acyltransferase
ELOVL2: ELOVL fatty acid elongase 2
PDK4: Pyruvate dehydrogenase kinase 4
PMP2: FABP9, fatty acid binding protein 9
FABP1: Fatty acid binding protein 1
LAMP3: Lysosomal associated membrane protein 3
SLC2A1: Solute carrier family 2 member 1
PCNA: Proliferating cell nuclear antigen
LPL: Lipoprotein Lipase
ADIPO: Adiponectin, C1Q and collagen domain containing
PPARγ: Peroxisome proliferator-activated receptor gamma
C/EBPβ: CCAAT/enhancer binding protein beta
HNF4A: Hepatocyte nuclear factor 4 alpha
SLC2A2: Solute carrier family 2 member 2
FABP7: Fatty acid binding protein 7
