# Supplementary material for: ALKBH5 regulates chicken adipogenesis by mediating LCAT mRNA stability depending on m6A modification
Source: BMC Genomics. 2024 Jun 25;25:634. doi: 10.1186/s12864-024-10537-2 (PMC11197345; doi:10.1186/s12864-024-10537-2)
Supplement: Supplementary file 1 — Supplementary Material 1 [file 12864_2024_10537_MOESM1_ESM.docx]

Table S1. The qPCR primer sequence information.

| Primers’ name | Primer sequences (5’ to 3’) | Product length（bp） | Annealing temperature（℃） |
| --- | --- | --- | --- |
| PPARγ-F | CTCCTTCTCCTCCCTATTT | 227 | 58 |
| PPARγ-R | TTTCTTATGGATGCGACA |  |  |
| Cyclin D1-F | CAGAAGTGCGAAGAGGAAGT | 188 | 58 |
| Cyclin D1-R | CTGATGGAGTTGTCGGTGTA |  |  |
| Cyclin D2-F | AACTTGCTCTACGACGACC | 150 | 58 |
| Cyclin D2-R | TTCACAGACCTCCAACATC |  |  |
| PCNA-F | GTGCTGGGACCTGGGTT | 217 | 58 |
| PCNA-R | CGTATCCGCATTGTCTTCT |  |  |
| Cyclin B2-F | CAGTAAAGGCTACGAAAG | 133 | 58 |
| Cyclin B2-R | ACATCCATAGGGACAGG |  |  |
| LPL-F | CCAAGGTAGACCAGCCATTC | 154 | 60 |
| LPL-R | TGCTCCAGGCACTTCACA |  |  |
| ADIPOR1-F | GACAAGAACAGCAACGAGTACCGC | 110 | 60 |
| ADIPOR1-R | CCTGAAGATGCCCCGCAGAGT |  |  |
| C/EBPβ-F | GCGGACTGTTTGGCTGCTCT | 220 | 60 |
| C/EBPβ-R | CGGGTGAGGCTGATGTAGGTGT |  |  |
| LCAT-F | GTCCTGGCGTCAGGTGATAAT | 183 | 62 |
| LCAT-R | GAAGAACCGCTGGTAGTCCC |  |  |
| GAPDH-F | CAACTTTGGCATTGTGGAGG | 130 | 56~62 |
| GAPDH-R | CGCTGGGATGATGTTCTGG |  |  |

Table S2. The sequence of the predicted loci.

| Name | Sequences |
| --- | --- |
| *LCAT*-1 | AAGGAGGCGATGGCCAGCTTCCCTCCATGCTTGTATCCACCAGAAGCTGGCgTGAAGAGGAGTAAAGGACTTGGAGtTGGGTATCAGAGAGAAGGAGCAAGAACAAC |
| *LCAT*-2 | ACTTGCTGAAGGGATTACCCCCTCCTGGTGTGGACACATATTGCCTTTATGGCACGGGCTATCCCACAGTGGAGACTTACATATACGATGAGCATTTCCCTTACGAGGACCCCGTGGACATGATTTATGGTGATGGGGACGACACTGTCAACA |

Table S3. Primer information for MeRIP-qPCR

| Primers’ name | Primer sequences (5’ to 3’) | Product length（bp） | Annealing temperature（℃） |
| --- | --- | --- | --- |
| RIP-*LCAT*-1-F | GCTTCCCTCCATGCTTGTAT | 83 | 58 |
| RIP-*LCAT*-1-R | TGCTCCTTCTCTCTGATACCC |  |  |
| RIP-*LCAT* -2-F | ACTTGCTGAAGGGATTACC | 153 | 60 |
| RIP -*LCAT*-2-R | TGTTGACAGTGTCGTCCCC |  |  |

Table S4. Statistical analysis of sequence alignment between sequencing data and reference.

| Sample | Raw Reads | Clean Reads | Clean Read Q30 Rate | Mapped Reads | Mapped Rate |
| --- | --- | --- | --- | --- | --- |
| L1-IN | 66,201,858 | 66,020,700 | 97.74% | 61,774,540 | 93.57% |
| L2-IN | 68,353,460 | 68,180,342 | 97.95% | 64,173,405 | 94.12% |
| L3-IN | 66,846,292 | 66,550,794 | 97.65% | 61,432,098 | 92.31% |
| H1-IN | 63,886,012 | 63,672,604 | 97.71% | 59,597,043 | 93.60% |
| H2-IN | 59,461,434 | 59,244,038 | 98.00% | 55,819,574 | 94.22% |
| H3-IN | 64,739,680 | 64,547,770 | 97.98% | 60,674,131 | 94.00% |
| L1-IP | 78,140,778 | 75,560,930 | 97.64% | 67,184,757 | 88.91% |
| L2-IP | 70,291,004 | 68,794,656 | 97.48% | 59,740,989 | 86.84% |
| L3-IP | 68,366,508 | 67,143,156 | 97.34% | 58,217,166 | 86.71% |
| H4-IP | 69,194,202 | 67,289,956 | 97.74% | 60,921,666 | 90.54% |
| H5-IP | 73,421,796 | 72,157,096 | 97.70% | 62,077,230 | 86.03% |
| H6-IP | 80,297,036 | 78,802,210 | 97.70% | 66,246,133 | 84.07% |

Table S5. m^6^A enrichment area statistics.

| Sample | nPeak | Length | Mean Length |
| --- | --- | --- | --- |
| L1 | 28,229 | 28,279,745 | 1001.8 |
| L2 | 30,287 | 33,792,012 | 1115.73 |
| L3 | 25,619 | 23,180,444 | 904.81 |
| H1 | 25,522 | 23,004,711 | 901.37 |
| H2 | 17,264 | 18,602,371 | 1077.52 |
| H3 | 17,008 | 14,374,646 | 845.17 |
