# Supplementary figures and images for: ALKBH5 regulates chicken adipogenesis by mediating LCAT mRNA stability depending on m6A modification
Source: BMC Genomics. 2024 Jun 25;25:634. doi: 10.1186/s12864-024-10537-2 (PMC11197345; doi:10.1186/s12864-024-10537-2)

Fig S1.: The result of flow cytometry.


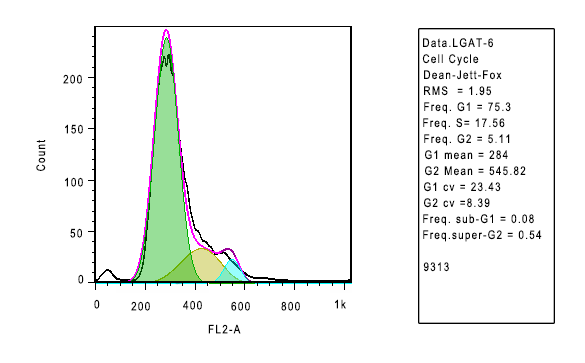

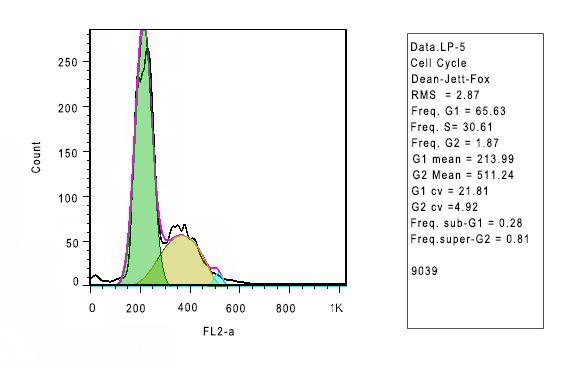


pcDNA3.1

LCAT

Supplement: Supplementary file 2 — Supplementary Material 2 [file 12864_2024_10537_MOESM2_ESM.docx]
