# Supplementary material for: ALKBH5 regulates chicken adipogenesis by mediating LCAT mRNA stability depending on m6A modification
Source: BMC Genomics. 2024 Jun 25;25:634. doi: 10.1186/s12864-024-10537-2 (PMC11197345; doi:10.1186/s12864-024-10537-2)
Supplement: Supplementary file 3 — Supplementary Material 3 [file 12864_2024_10537_MOESM3_ESM.docx]

**(A)** Original image of m^6^A methylation modification after *ALKBH5* overexpression in ICP cells. The membrane is not cut prior to hybridisation with the antibody.


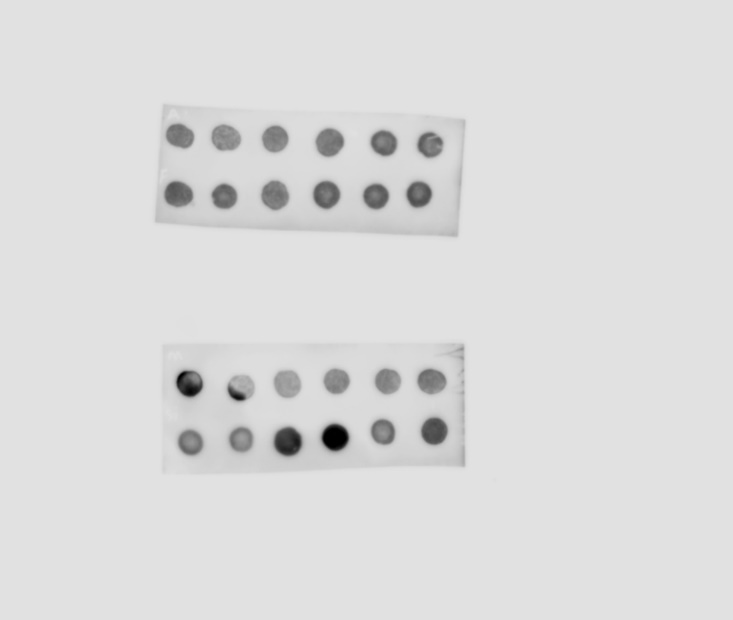


**A**

**ALKBH5**

**pcDNA3.1-flag**

**m^6^A**

**FTO**

**pcDNA3.1-flag**

**m^6^A**

**FTO**

**pcDNA3.1-flag**

**METTL3**

**pcDNA3.1-flag**

**ALKBH5**

**25kDa**

**38kDa**


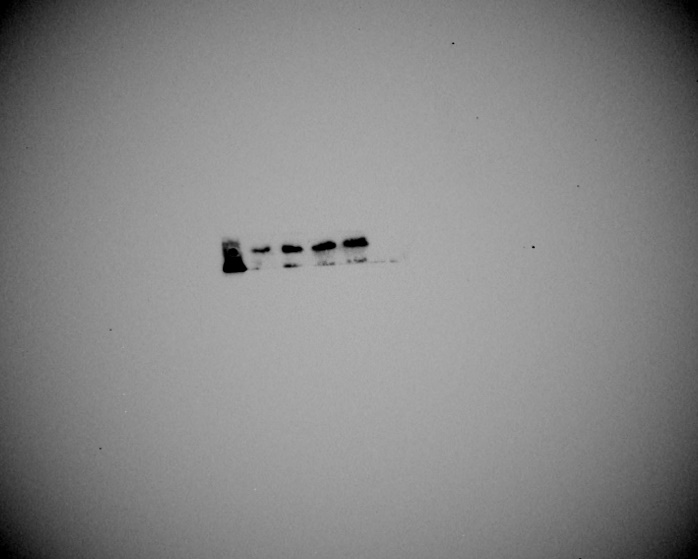


**B**

**ALKBH5**

**HF**

**LF**

**44kDa**

**Marker**

**GAPDH**

**36 kDa**


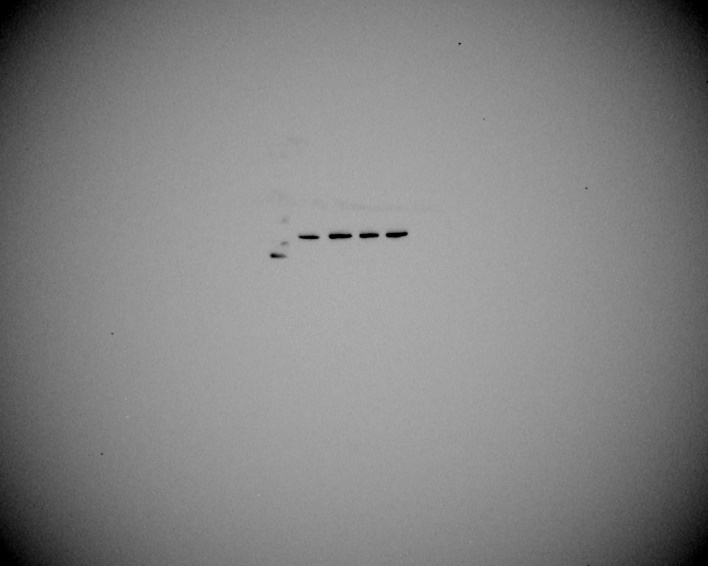


**Marker**

**LF**

**HF**

**(B)** The western blot for *ALKBH5* protein in HF and LF, The membrane is sheared before hybridisation with the antibody.the western blot membrane were cropped at 38kD.

**25kDa**


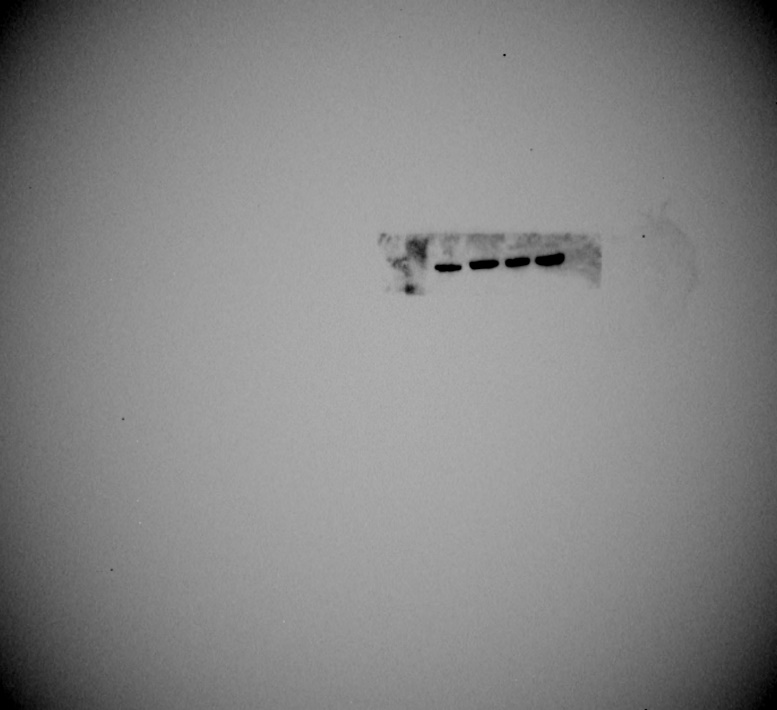

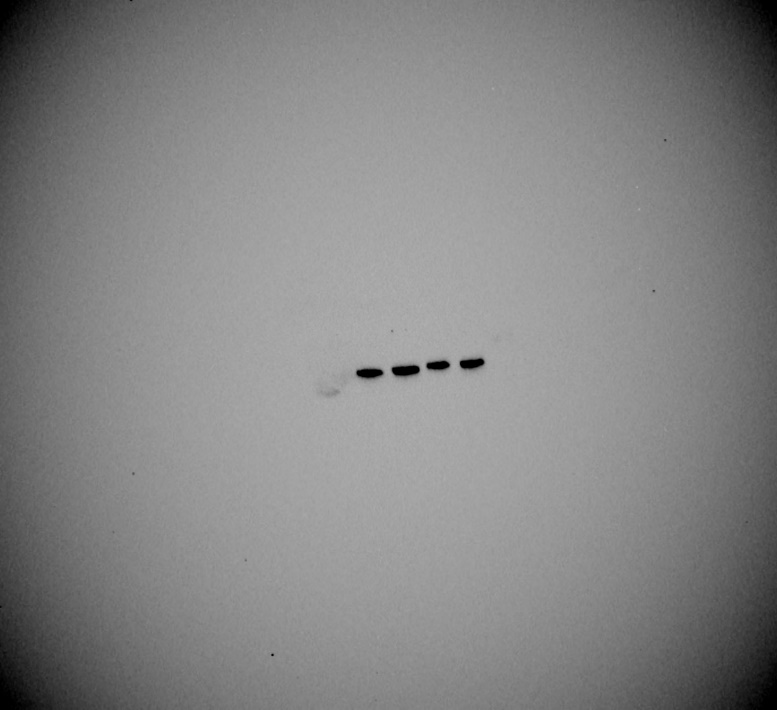


**C**

**LCAT**

**HF**

**LF**

**60 kDa**

**Marker**

**GAPDH**

**36 kDa**

**Marker**

**LF**

**HF**

**(C)** The western blot for *LCAT* protein in HF and LF. The membrane is sheared before hybridisation with the antibody.the western blot membrane were cropped at 38kD.

D


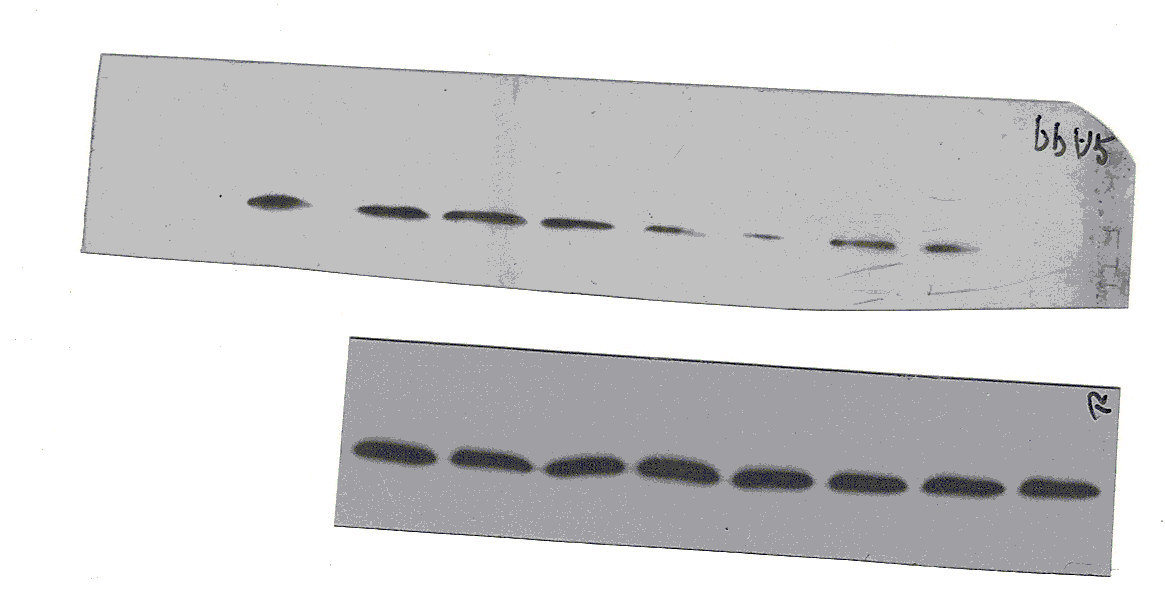


**PPARγ**

**GAPDH**

**54 kDa**

**36 kDa**

**si-NC**

**si-LCAT**

**LCAT-pcDNA3.1**

**pcDNA3.1**

**(D)** The western blot for *PPARγ* protein after overexpression and interference with *LCAT*. The image is an x-ray exposure. The membrane is sheared before hybridisation with the antibody.the western blot membrane were cropped at 38kD.
